# Analyzing aberrant DNA methylation in colorectal cancer uncovered intangible heterogeneity of gene effects in the survival time of patients

**DOI:** 10.1038/s41598-023-47377-1

**Published:** 2023-12-13

**Authors:** Saeedeh Hajebi Khaniki, Farhad Shokoohi, Habibollah Esmaily, Mohammad Amin Kerachian

**Affiliations:** 1 Department of Biostatistics, School of Health, Mashhad University of Medical Sciences, Mashhad, Iran; 2 Department of Mathematical Sciences, University of Nevada Las Vegas, Las Vegas, NV 89154 USA; 3 Social Determinants of Health Research Center, Mashhad University of Medical Sciences, Mashhad, Iran; 4 Medical Genetics Research Center, Mashhad University of Medical Sciences, Mashhad, Iran

**Keywords:** Cancer epigenetics, Statistical methods

## Abstract

Colorectal cancer (CRC) involves epigenetic alterations. Irregular gene-methylation alteration causes and advances CRC tumor growth. Detecting differentially methylated genes (DMGs) in CRC and patient survival time paves the way to early cancer detection and prognosis. However, CRC data including survival times are heterogeneous. Almost all studies tend to ignore the heterogeneity of DMG effects on survival. To this end, we utilized a sparse estimation method in the finite mixture of accelerated failure time (AFT) regression models to capture such heterogeneity. We analyzed a dataset of CRC and normal colon tissues and identified 3406 DMGs. Analysis of overlapped DMGs with several Gene Expression Omnibus datasets led to 917 hypo- and 654 hyper-methylated DMGs. CRC pathways were revealed via gene ontology enrichment. Hub genes were selected based on Protein–Protein-Interaction network including *SEMA7A*, *GATA4*, *LHX2*, *SOST*, and *CTLA4*, regulating the Wnt signaling pathway. The relationship between identified DMGs/hub genes and patient survival time uncovered a two-component mixture of AFT regression model. The genes *NMNAT2*, *ZFP42*, *NPAS2*, *MYLK3*, *NUDT13*, *KIRREL3*, and *FKBP6* and hub genes *SOST*, *NFATC1*, and *TLE4* were associated with survival time in the most aggressive form of the disease that can serve as potential diagnostic targets for early CRC detection.

## Introduction

Colorectal cancer (CRC), the third most common cancer worldwide, is a group of diseases characterized by genetic and epigenetic changes^1,2^. Despite being the second leading cause of cancer-related deaths, less attention has been paid to early detection due to the fact that patients do not adhere to invasive screening tests such as colonoscopy^3^. It has been shown that epigenetic alterations in solid and liquid biopsies can be used for early detection and thus prognosis and effective treatment^4^. DNA methylation at CpG sites (5mc) is an epigenetic mark that regulates gene expression through transcriptional silencing^5^. Aberrant DNA methylation plays a crucial role in the pathogenesis and progression of CRC and has emerged as a promising diagnostic marker for the disease^6^. In particular, aberrant DNA methylation can impact genes where their inactivation may exacerbate tumor formation through the induction of genomic instability or by directly silencing the methylated gene^7^.

Much research has been done to develop comprehensive panels of biomarkers based on DNA methylation that can facilitate accurate diagnosis of CRC^8^. While the genes *SEPT9, NDRG4*, and *BMP3* are FDA-approved for CRC^9,10^, there are many other genes such as *APC, SFRP1, TFPI2*, and *VIM* that have not yet been approved^8^.

In order to detect and validate genes that are potential CRC biomarkers, the following steps should be taken. Firstly, a panel of biomarkers must be developed using accurate statistical methods with a deep understanding of the underlying biology of the disease and the molecular mechanisms that drive them. Secondly, the significant biomarkers must be validated via in silico validation using several other datasets; and thirdly, the effectiveness of top candidate biomarkers in improving patient health should be verified using survival models. Lack of adequate precision in each of the above steps leads to misleading conclusions. Among others, two issues affect precision: removing genomic positions with missing values or low read-depth and ignoring the heterogeneity of DMG effects on survival times.

To accurately predict the differentially methylated profiles in CRC, one must consider all biological and environmental factors such as dietary^11^, aging^12^, and hazardous behaviors^13^ (e.g., smoking), among others. Such factors are often ignored by most studies when predicting methylation profiles. In addition, methylation data always suffer from heavy missing values that can affect subsequent analyses. For instance, 68% of CpG sites have missing values in at least one sample in our dataset (Section 2). Almost all DNA methylation pipelines, except a few such as the DMCHMM method^14^, filter out such positions from the analysis. We used DMCHMM to not only account for extra covariates but also efficiently impute the missing values.

Having identified the differentially methylated genes (DMG) associated with CRC and validating them, it is crucial to identify their underlying signaling pathways that regulate gene expression^15,16^. The main known CRC pathways are Wnt^17^, MAPK^18^, TGF-$\beta$^19^, and TP53^20^. Although significant progress has been made in understanding the biology of CRC, there are still many unknown pathways and mechanisms involved in this disease. Identification of hub genes, also known as driver genes is the next step in the analysis of biomarker detection. Hub genes play a critical role in regulating several genes in the biological network and have the potential to be regarded as therapeutic targets in CRC^21^.

In the next step, the relationship between identified DMGs and the survival time of CRC patients should be evaluated. Most studies employ a limited panel of biomarkers selected through conventional univariate Cox proportional hazard regression models and overlook the potential effects of the rest of the biomarkers^22–24^. In a recent study^25^, the Cox-LASSO survival model was used to account for a larger set of biomarkers but ignored the heterogeneity of covariate effects. To the best of our knowledge, none of the studies have taken into account the heterogeneity of DMG effects on survival time. To address this problem, one may use the sparse estimation method in the finite mixture of accelerated failure time (AFT) regression models^26^. Prior to this step, it is common to screen the number of genes to a manageable magnitude. This process can be done by selecting the top highly correlated genes with survival time of the patients using the correlation-adjusted scoring method^27^.

This study aimed to identify CRC-related DMGs to serve as potential biomarkers for early detection by including all the available information in the data and avoiding the exclusion of any genomic position. To this end, we acquired a high-throughput DNA methylation dataset which consists of patients with CRC and healthy individuals. Information on age, history of smoking, and drug abuse was also collected. A description of the data is provided in Section “Methods”. Information on other datasets used for validation and survival analysis and all statistical and Bioinformatics methods are listed in this section. In Section “Results”, a comprehensive analysis of data is conducted. Section “Discussion” gives a discussion and some concluding remarks.

## Methods

**Figure 1:**
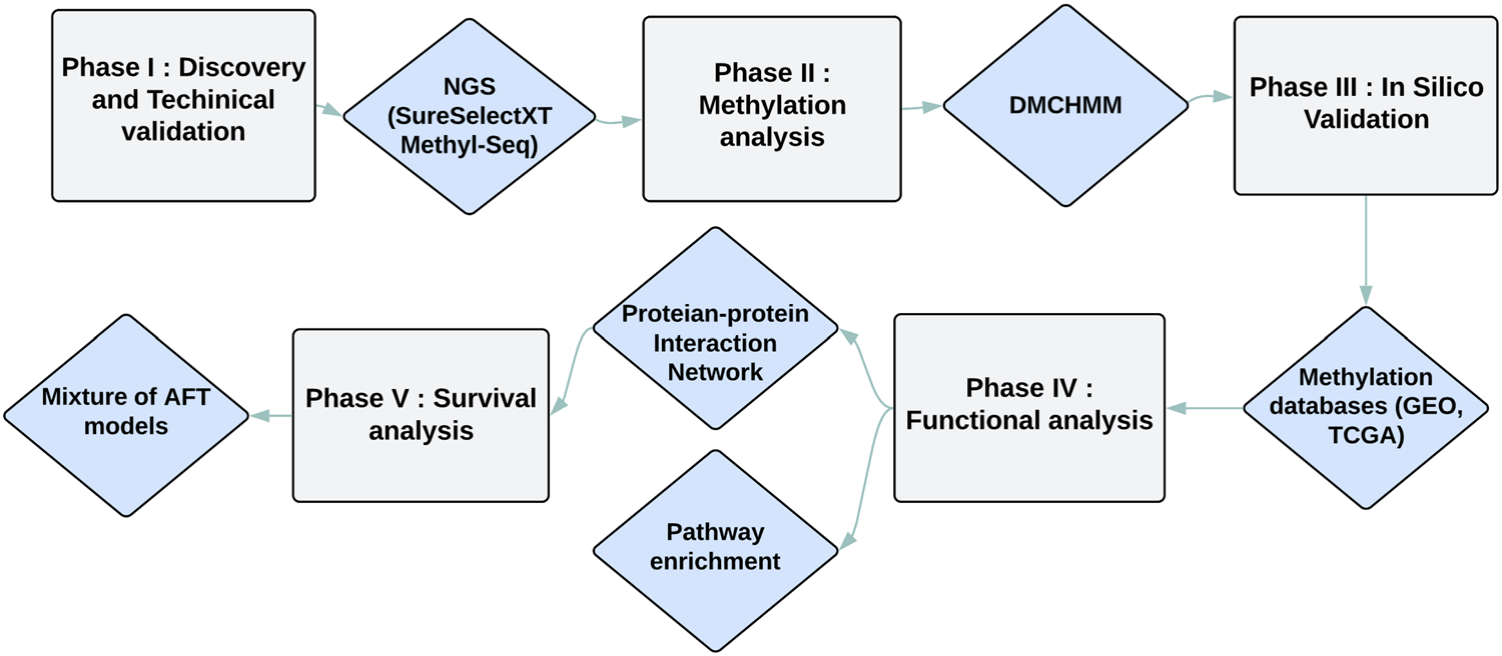
Study workflow for the analysis of CRC datasets.

In this section, we outline the data analysis process we followed to detect DMGs, hub genes, and their effects on the survival time and enriched pathways of CRC. Figure 1 depicts the flowchart of this process.

### Phase I (pre-processing of discovery samples)

To identify methylation-based CRC biomarkers, information on 6 patients with adenocarcinoma of CRC and 6 normal males was obtained. Two groups were matched based on age, and family history of cancer^28^.

The methylation profiles in our dataset are derived from a three-step pre-processing phase conducted through SureSelectXT Human Methyl-Seq. Initially, the purity and quantity of 12 DNA tissue samples were assessed using specific criteria, including a minimum concentration of $50 ng/\upmu {\text{l}}$, a purity ratio $(A260/A280) \ge 1.7$, a volume of at least $20 ng/\upmu {\text{l}}$, and a total amount exceeding $3.0 \upmu {\text{g}}$. Subsequently, global methylation profiles of CRC and normal samples were analyzed using SureSelectXT Human Methyl-Seq.

Pre-sequencing tasks, such as sample collection and DNA extraction, were consistently carried out by a single technician. Experimental conditions for all samples remained constant both before and after sequencing. During the sequencing process, all sample runs were executed simultaneously using the same device, employing Next-generation sequencing technology, a highly parallel sequencing method. This approach minimized the potential introduction of batch effects attributable to non-biological factors such as variations in laboratory conditions, personnel, and equipment used in the experiment.

In the second step, a quality control assessment of total reads using FastQ^29^ was conducted. This step aimed to provide informative global and graphical representations of read quality in methylation sequencing, both pre and post-alignment. Notably, our data consistently exhibited high quality in raw sequencing reads across all samples. Subsequently, Trim Galore^30^ was utilized to process the raw sequencing reads. This involved the removal of sequencing adapters, specifically the Illumina universal adapter, and discarding the low-quality bases (those with quality scores below 67, as per Illumina standards) located at the 3ʹ end of reads. Additionally, any ambiguous bases found in both reads were removed.

Finally, the raw bisulfite sequencing data were aligned to the human reference genome (GRCh37/19) using Bismark^31^. Several comparisons and visualization confirmed minimal to no presence of batch effects in our data. This discovery dataset includes methylation read counts and read-depth for each CpG site, generating 57 to 76 million Illumina sequencing reads per subject. Between 88.5% and 89.8% of sequenced reads were mapped to either strand of the human genome (GRCh37/19). On average, each CpG site was sequenced between 19 × and 24 × per sample. The sequencing details for the subjects are presented in Table 1. Approximately 68% of the 19,530,818 CpG sites have missing information in at least one sample.

**Table 1:** Summary statistics of methylation sequencing reads of discovery samples.

Sample	Total reads	Mapping rate	Methylation (%)	Average coverage	GC (%)
T65	76,723,684	88.50	47.70	24.15	27.04
N16	70,443,130	88.70	45.70	23.53	27.26
T20	67,394,464	88.90	44.70	19.58	27.03
N4	68,165,382	88.80	46.50	22.19	27.19
T31	61,789,306	89.00	46.90	21.69	26.92
N10	57,311,634	89.05	46.70	19.26	27.04
T35	79,004,644	88.90	46.10	24.43	27.11
N7	75,663,274	89.00	47.20	22.62	27.04
T45	64,188,480	89.00	47.40	21.22	27.06
N8	57,091,968	89.80	46.80	20.42	27.41
T67	61,203,576	89.30	44.30	20.77	27.17
N14	66,871,860	89.60	47.40	22.17	27.11

### Phase II (identification of differentially methylated genes)

We utilized the DMCHMM pipeline^32^ to identify CpGs with differentially methylated patterns between CRC and normal discovery samples. We specifically did not remove any position with missing information or low read-depth. The missing information was imputed using DMCHMM via hidden Markov models^14^. Significant differentially methylated cytosines (DMCs) were selected based on the FDR threshold of 0.05. DMCs were aligned to the human reference genome (GRCh37/19) using the UCSC Genome Browser (https://genome.ucsc.edu). A gene whose promoter was mainly hypo- or hyper-methylated was classified as hypo- or hyper DMG, respectively.

### Phase III (cross-platform validation)

To validate our result, several methylation profiles (GSE53051^33^, GSE77718^34^, GSE101764^13^, GSE42752^35^, GSE48684^36^) were extracted from the Gene Expression Omnibus (GEO, https://www.ncbi.nlm.nih.gov/geo/). Of these datasets, a total of 212 CRC and 242 normal mucosa tissue samples were selected based on setup conditions to minimize the confounding effect of other variables. These datasets have provided valuable insights into the molecular alterations that occur in CRC, and their findings have implications for the diagnosis and treatment of this disease. For the analysis of methyl array profiles of validation sets, the GEO2R (http://www.ncbi.nlm.nih.gov/geo/geo2r/) web tool and the limma R-package^37^ were used. To mitigate batch effects, we applied the ‘removeBatchEffect’ option from the package. A probe was considered differentially methylated if its adjusted p-value was less than 0.05, and the absolute of $\log _2$ of methylation fold change was greater or equal to 1. The differentially methylated probes were aligned to the human reference genome (GRCh37/19) using the FDb.InfiniumMethylation.hg19 package^38^. In the last step, we compared the lists of DMGs based on the validation sets and our discovery samples to identify consistent hypo/hyper-methylated genes across different populations and platforms.

### Phase IV (network construction and functional analysis)

In order to investigate the Protein-Protein Interaction (PPI) network and module analysis, we utilized the ‘Search Tool for the Retrieval of Interacting Genes’ (STRING) database. We set the interaction score threshold to 0.4 to screen for high-confidence interactions and visualized the resulting network using the Cytoscape^39^ software (Version 3.9.1). Next, we employed the Molecular Complex Detection (MCODE) algorithm to uncover densely connected substructures within the network. The MCODE score must be greater than 3 and the minimum number of nodes must be 4. In order to identify key hub genes within the network, we used the cytoHubba plugin and considered the degree of centrality as a parameter.

To gain insight into the biological mechanisms that are driving CRC and prioritize identified DMGs, we performed functional and pathway enrichment analysis using DAVID^40^ (https://david.ncifcrf.gov/). Gene ontology (GO) terms and Kyoto Encyclopedia of Genes and Genomes (KEGG^41^) pathways were considered significantly enriched if the p-values were less than 0.05 and the q-values were less than 0.1. The visualization of the identified GO terms and KEGG pathways were done with the clusterProfiler^42^, pathfindR^43^, and ShinyGO^44,45^ (http://bioinformatics.sdstate.edu/go/) packages.

### Phase V (Uncovering intangible heterogeneity of DMG effects on survival time)

To explore the relationship between identified DMGs and survival time, the DNA methylation profiles of 521 samples were obtained from The Cancer Genome Atlas (TCGA) network^46^. Complete information on clinical variables including days to follow-up and the status of the patient were analyzed.

**Figure 2:**
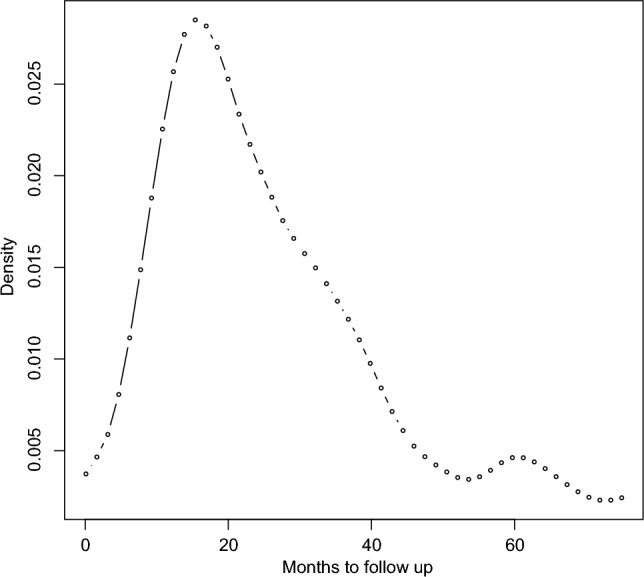
Density estimation of overall survival time (in months) in CRC patients (R package survPresmooth, v1.1-11).

We conducted several preliminary analyses on the overall survival time of patients with CRC. First, we estimated the density of the logarithm of the survival times using the Kaplan-Meier estimator. The density plot in Figure 2 shows a mixture distribution. Second, we applied mixture and non-mixture models of normal distributions using the mixtools package^47^. The BICs for the mixture of components $K=1, 2, 3, 4$, and 5 were estimated as 777.73, 709.02, 712.31, 722.51, and 721.68, respectively, with the lowest BIC observed for the mixture with 2 components. Finally, we employed mixture and non-mixture models of semiparametric scaled data using the stochastic EM algorithm^48^ via mixtools. Please note that the package only outputs a mixture with two components, and the BICs for the non-mixture $K=1$ and the mixture of 2 components were 1323.62 and 582.01, respectively. Similarly, the lowest BIC was observed for the mixture with 2 components. All of these preliminary analyses indicate heterogeneity in the overall survival time of patients with CRC.

Furthermore, a comprehensive literature review revealed numerous conflicting results. For example, researchers^49^ found a significant association between the methylation level of RASSF1A and the overall survival of CRC patients, while other studies^50^ did not observe such an association.

Moreover, several studies indicate stage-specific^51^ and age-specific^52^ effects of DNA methylation in certain genes on the survival outcomes of CRC patients. These results clearly suggest heterogeneity in the overall survival time of patients with CRC.

We hypothesize that the effect of identified DMGs and hub genes on the overall survival time varies in each subpopulation, but not all DMGs and hub genes have an effect in each subpopulation, implying that the underlying regression model is sparse. To capture such heterogeneity, we employed the sparse estimation method in the finite mixture of AFT regression models^26^. The details of the method are given in Section “Sparse finite mixture of AFT regression models to estimate the DMG effects on the survival times” below. The response variable is “Overall Survival Time”, and the independent variables are the log-transformed average methylation of identified DMGs or hub genes that we discovered through Phase II–IV. The goal of such a regression model is to estimate the effects of each gene in different sub-populations of the response variable, providing insights into the effects of each gene on the survival time of patients with CRC. It is important to note that the response variable (survival time) is subject to right-censoring. The sparse estimation method requires tuning parameters, which are estimated using a data-adaptive approach explained in Section “Sparse finite mixture of AFT regression models to estimate the DMG effects on the survival times”.

### Sparse finite mixture of AFT regression models to estimate the DMG effects on the survival times

As hypothesized above, the overall survival time of patients with CRC is heterogeneous; thus, we hypothesize that the relationship between overall survival time and DMGs and hub genes found in Phase II-IV is heterogeneous. Such heterogeneity cannot be detected using a regular AFT regression model for censored data. Therefore, we employ the finite mixture of the AFT regression model to capture intangible DMG and hub gene effects on survival time. To this end, we use the finite mixture of AFT regression model:

$$f(y; \varvec{\theta }) \propto \sum \nolimits _{k=1}^{K} \pi _k \left[ f_k(y; \textbf{X} \varvec{\beta }_k, \sigma ^2_k)\right] ^\delta \left[ S_k(y; \textbf{X} \varvec{\beta }_k, \sigma ^2_k)\right] ^{1-\delta },$$

where $f_k$ and $S_k$ are respectively the density of normal distribution and its survival function, $y = \log (t)$, *t* is the overall survival time, $\delta$ is an indicator representing right-censored (i.e., $\delta = 0$ if time is censored and 1 if it is not censored), $\textbf{X}$ is the vector of all DMGs and hub genes discovered in Phase II-IV, $\varvec{\beta }_k$ is the vector of effects of these genes in Component *k* of the mixture model, $\sigma _k^2$ is the variance, and $\pi _k$ is the proportion of the *k*th component.

It is common to screen the number of genes prior to analysis in case of a large number of identified genes. To this end, we applied a correlation-adjusted score method using the carSurv package^27^ to screen the genes.

Next, we used the fmrs package^53^ to fit finite mixture and non-mixture of AFT regression models to the data. We employed the smoothly clipped absolute deviations (SCAD) penalty^26^. This sparse method requires *K* tuning parameters which are estimated via the data-adaptive component-wise BIC method proposed in Shokoohi et al.^26^.

## Results

### Differentially methylated cytosine detection

**Figure 3:**
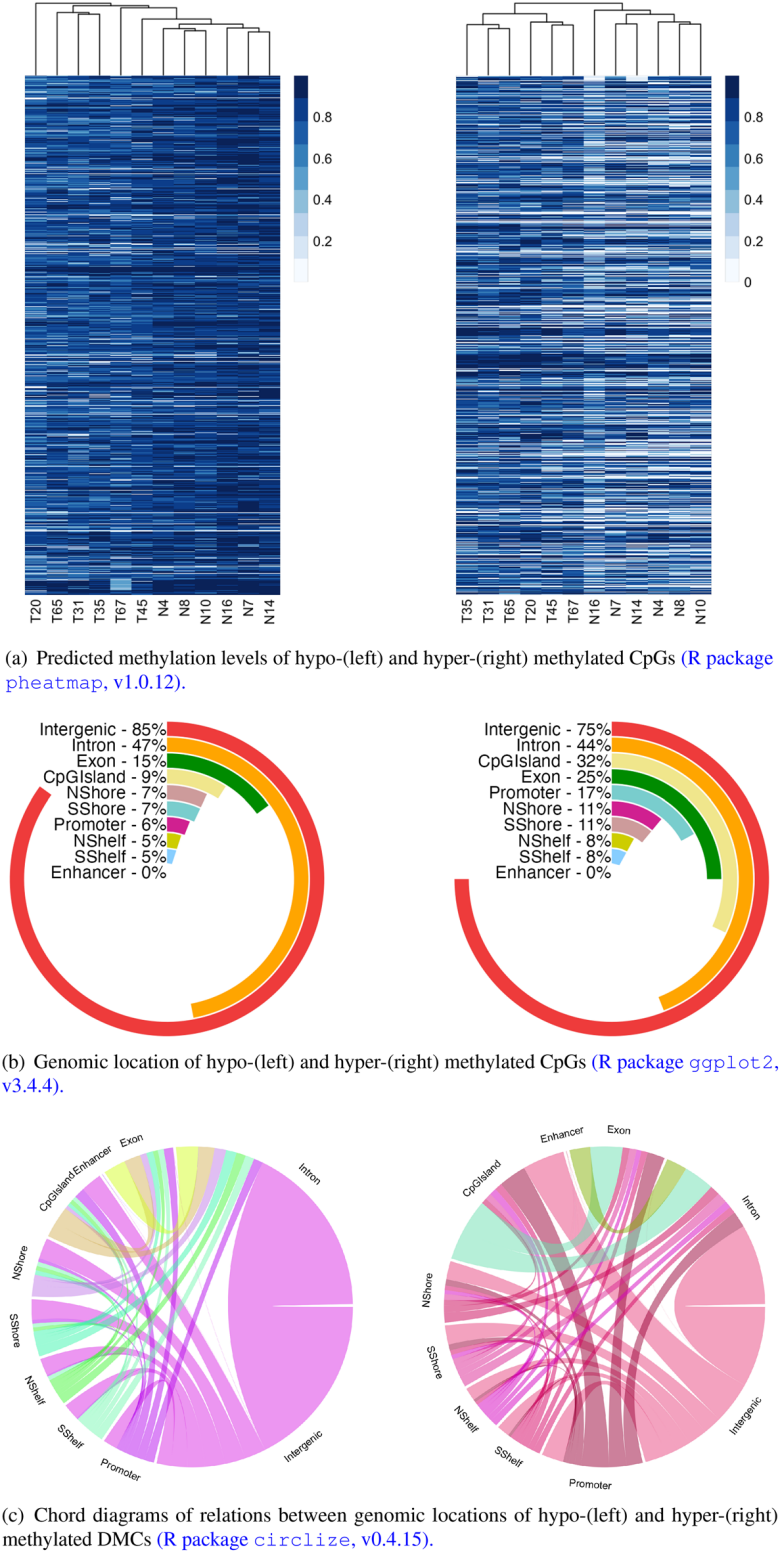
Genomic location of identified differentially methylated CpGs and their predicted levels in CRC (T) and normal (N) samples using DMCHMM. The hierarchical clustering of CRC and normal samples in the heatmaps is based on complete linkage.

We identified 2,691,019 DMCs between CRC and normal groups of the discovery dataset while adjusting for the potential confounding effect of smoking history or drug abuse. Of these identified DMCs, 1,985,557 positions were hypo-methylated and 705,462 CpGs were hyper-methylated in CRC vs normal samples. The heatmaps (see R package pheatmap^54^) in Fig. 3a indicate a clear clustering pattern between the CRC and normal samples based on the predicted methylation levels of DMCs.

To explore the genomic location of the DMCs, we analyzed their distribution across different regions and summarized the results in Fig. 3b. Intergenic regions were found to harbor the majority of the detected DMCs both in the hypo and hyper categories. Notably, we observed that 32% of hyper-methylated DMCs were located in CpG islands, while only 9% of hypo-methylated DMCs were found in these regions. Additionally, the regions with the highest percentage of hyper-methylated DMCs were identified in introns, exons, and CGI shores. The Chord diagrams (see R package circlize^55^) in Fig. 3c gives a comprehensive overview of how hyper and hypo-methylated DMCs were distributed across different genomic regions. Our findings suggest that many DMCs in intergenic regions were expanded to intronic regions in both hypo and hyper-methylated categories.

Given the potential significance of promoter methylation in cancer development and progression, we focused our subsequent analysis on DMCs located on gene promoters, which encompassed 268,978 CpGs. These CpGs resided on 3406 gene promoters, of which 1394 were hyper-methylated and 2012 were hypo-methylated. The list of DMGs is available as supplementary material.

#### Robust DMGs in CRC

**Figure 4:**
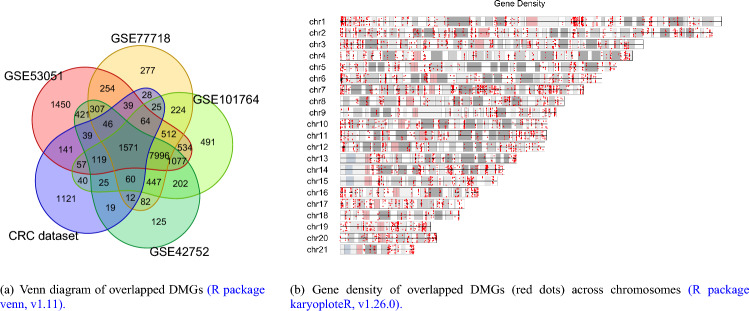
Summary of common identified DMG and their distribution.

To verify the robustness of identified DMGs, we performed a cross-platform procedure with DMGs identified in selected GEO datasets as depicted in Fig. 4a (see R package venn^56^). The comparison revealed a total of 1571 overlapped DMGs that were consistently identified across multiple studies. As Fig. 4b (see R package karyoploteR^57^) illustrated, the identified DMGs were spread almost evenly across different chromosomes, with chromosomes 1 and 7 having some dense regions of CRC-related DMGs. Within this set, 917 genes were hypo-methylated, and 654 genes were hyper-methylated. We focused our subsequent analysis on these identified DMGs to gain a deeper understanding of their role in CRC pathogenesis.

##### GO enrichment KEGG pathway analysis

**Figure 5:**
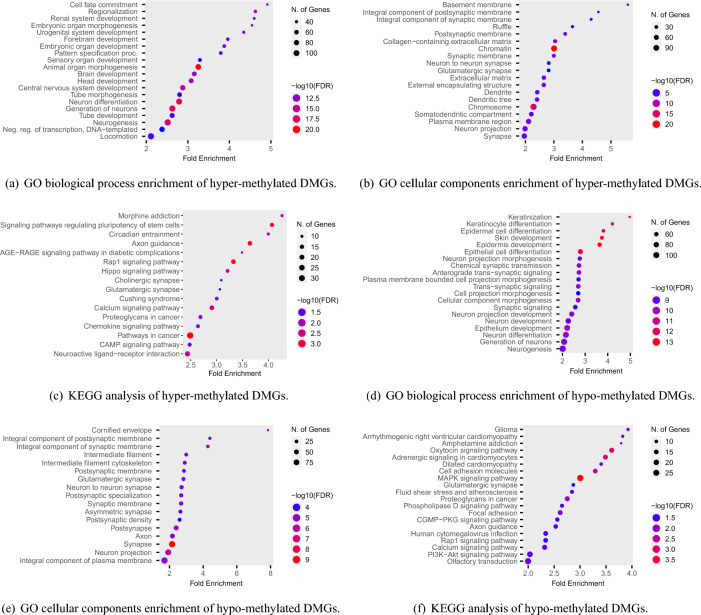
Enrichment analysis of commonly identified DMGs (R Shiny package ShineyGO, v0.77).

The analysis of robust DMGs in CRC utilizing the DAVID tool yielded a variety of enriched biological processes, molecular functions, and cellular components. Specifically, the hyper-methylated DMGs were found to be principally involved in ‘cell fate commitment’, ‘regionalization’, ‘embryonic organ morphogenesis’, ‘embryonic organ development’, ‘pattern specification process’, ‘animal organ morphogenesis’, ‘tube morphogenesis’, ‘tube development’, and ‘neurogenesis’ in the context of biological processes (Fig. 5a, see R Shiny package ShinyGO^44^). Enriched cellular components included ‘basement membrane’, ‘integral component of postsynaptic membrane’, and ‘Collagen-containing extracellular matrix’ (Fig. 5c). Additionally, KEGG pathway analysis indicated that hyper-methylated DMGs were significantly enriched in several pathways, including ‘signaling pathways regulating pluripotency of stem cells’, ‘axon guidance’, ‘morphine addiction’, ‘rap1 signaling pathway’, ‘circadian entrainment’, and ‘pathways in cancer’ (Fig. 5e and Table 2). Regarding biological processes, the hypo-methylated DMGs were found to be associated with a number of processes including ‘keratinization’, ‘keratinocyte differentiation’, ‘epidermal cell differentiation’, and ‘epithelial cell differentiation’ (Fig. 5b). Furthermore, analysis of the cellular component pathway revealed that the hypo-methylated DMGs were most significantly enriched in the ‘cornified envelope’, ‘integral component of the synaptic membrane’, and ‘integral component of the postsynaptic membrane’. Notably, these cellular components demonstrated the highest FDR and fold enrichment (Fig. 5d). Regarding molecular functions, the pathways with higher fold enrichment included ‘molecular transducer activity’, ‘signaling receptor activity’, and ‘transmembrane signaling receptor activity’. Notably, KEGG pathway analysis revealed that hypo-methylated DMGs were significantly enriched in several pathways, including the ‘oxytocin signaling pathway’, ‘glioma’, ‘adrenergic signaling in cardiomyocytes’, ‘MAPK signaling pathway’, ‘arrhythmogenic right ventricular cardiomyopathy’, and ‘cell adhesion molecules’ (Fig. 5f). These results offer valuable insights into the potential mechanisms of DMGs in CRC and identify possible therapeutic targets for this disease. A comprehensive summary of the KEGG pathways of hyper-methylated DMGs can be found in Table 2.

**Table 2:** KEGG pathway analysis of commonly identified hyper-methylated DMGs.

Enrichment		Pathway	Fold		
FDR	nGenes	Genes	Enrichment	Pathway	Matching proteins in network (labels)
0.0050	10	91	4.26	Morphine addiction	PDE8A, GNAS, SLC32A1, GABRA4, GNGT1, KCNJ3, ADORA1, ADCY1, PRKCB, GNG2
0.0004	15	143	4.07	Signaling pathways regulating pluripotency of stem cells	PAX6, FGFR1, LHX5, HOXA1, MYF5, WNT5A, ID2, BMP4, IGF1R, WNT3A, FZD1, FZD6, AXIN2, ONECUT1, SMAD2
0.0060	10	97	3.99	Circadian entrainment	GNAS, GNGT1, MTNR1B, ITPR1, KCNJ3, ADCY1, PRKCB, GRIN2A, PRKG1, GNG2
0.0004	17	181	3.64	Axon guidance	NEO1, PRKCZ, SEMA5B, NFATC2, CXCL12, UNC5A, WNT5A, EPHA4, SMO, EPHA7, SEMA4F, SEMA6D, SLIT2, ROBO3, UNC5C, SEMA4A, PLXNA4
0.0250	9	100	3.49	AGE-RAGE signaling pathway in diabetic complications	PRKCZ, STAT1, COL4A2, PLCD3, PRKCB, COL4A3, SMAD2, THBD, COL4A1
0.0006	18	210	3.32	Rap1 signaling pathway	PRKCZ, RASGRP2, APBB1IP, FGFR1, GNAS, FGF9, CNR1, VAV3, FGF5, IGF1R, ANGPT1, TIAM1, VAV2, ADCY1, PRKCB, ADORA2B, GRIN2A, SIPA1L1
0.0060	13	157	3.21	Hippo signaling pathway	CTNNA2, PRKCZ, FBXW11, TP73, WNT5A, ID2, BMP4, BMP6, WNT3A, FZD1, FZD6, AXIN2, SMAD2
0.0460	9	113	3.09	Cholinergic synapse	GNGT1, PIK3R5, ITPR1, KCNJ3, ADCY1, PRKCB, CHRM4, CHRM2, GNG2
0.0460	9	114	3.07	Glutamatergic synapse	GNAS, GNGT1, ITPR1, KCNJ3, ADCY1, PRKCB, GRIN2A, GNG2, GRM3
0.0180	12	155	3.00	Cushing syndrome	PDE8A, KCNK2, GNAS, CDK6, CRHR2, WNT5A, ITPR1, WNT3A, FZD1, ADCY1, FZD6, AXIN2
0.0030	18	240	2.91	Calcium signaling pathway	FGFR1, GNAS, FGF9, P2RX3, TACR1, FGF5, GNAL, ITPR1, PLCD3, ADCY1, PRKCB, GDNF, ADORA2B, OXTR, CHRM2, GRIN2A, ATP2A1, HRH1
0.0200	14	202	2.64	Chemokine signaling pathway	PRKCZ, RASGRP2, CXCL12, STAT1, PREX1, GNGT1, VAV3, PIK3R5, TIAM1, VAV2, ADCY1, PRKCB, GNG2
0.0500	11	166	2.56	Wnt signaling pathway	FBXW11, NFATC2, SFRP1, WNT5A, SFRP5, WNT3A, FZD1, SOX17, FZD6, PRKCB, AXIN2
0.0003	34	530	2.49	Pathways in cancer	CTNNA2, RASGRP2, FGFR1, GNAS, MSH2, FGF9, IL7, CDK6, CXCL12, WNT5A, STAT1, BMP4, GNGT1, SMO, RARA, CCNA1, COL4A2, LAMC1, FGF5, IGF1R, PMAIP1, WNT3A, FZD1, ADCY1, FZD6, PRKCB, AXIN2, COL4A3, SMAD2, GNG2, MITF, COL4A1, TXNRD1, NCOA4

##### PPI network construction

**Figure 6:**
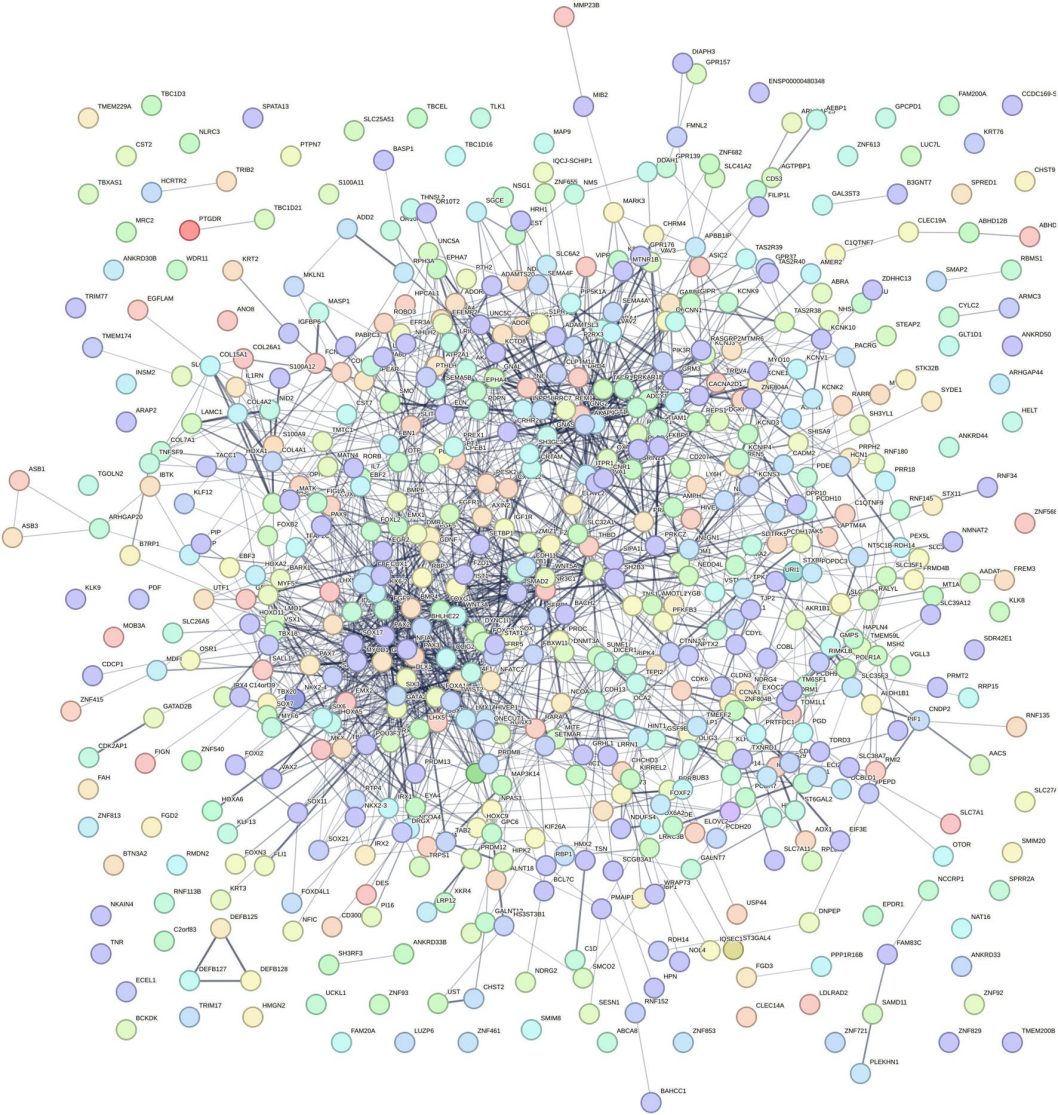
Protein–protein interaction network of hyper-methylated genes. Spots represent the proteins and lines show interactions (R Shiny package ShineyGO, v0.77).

**Figure 7:**
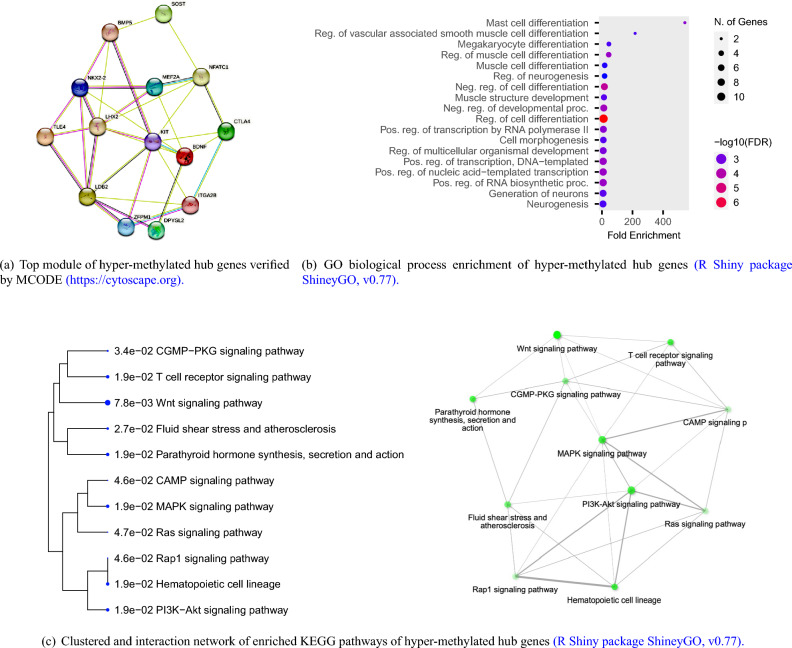
Bioinformatic analysis of hyper-methylated hub genes.

**Figure 8:**
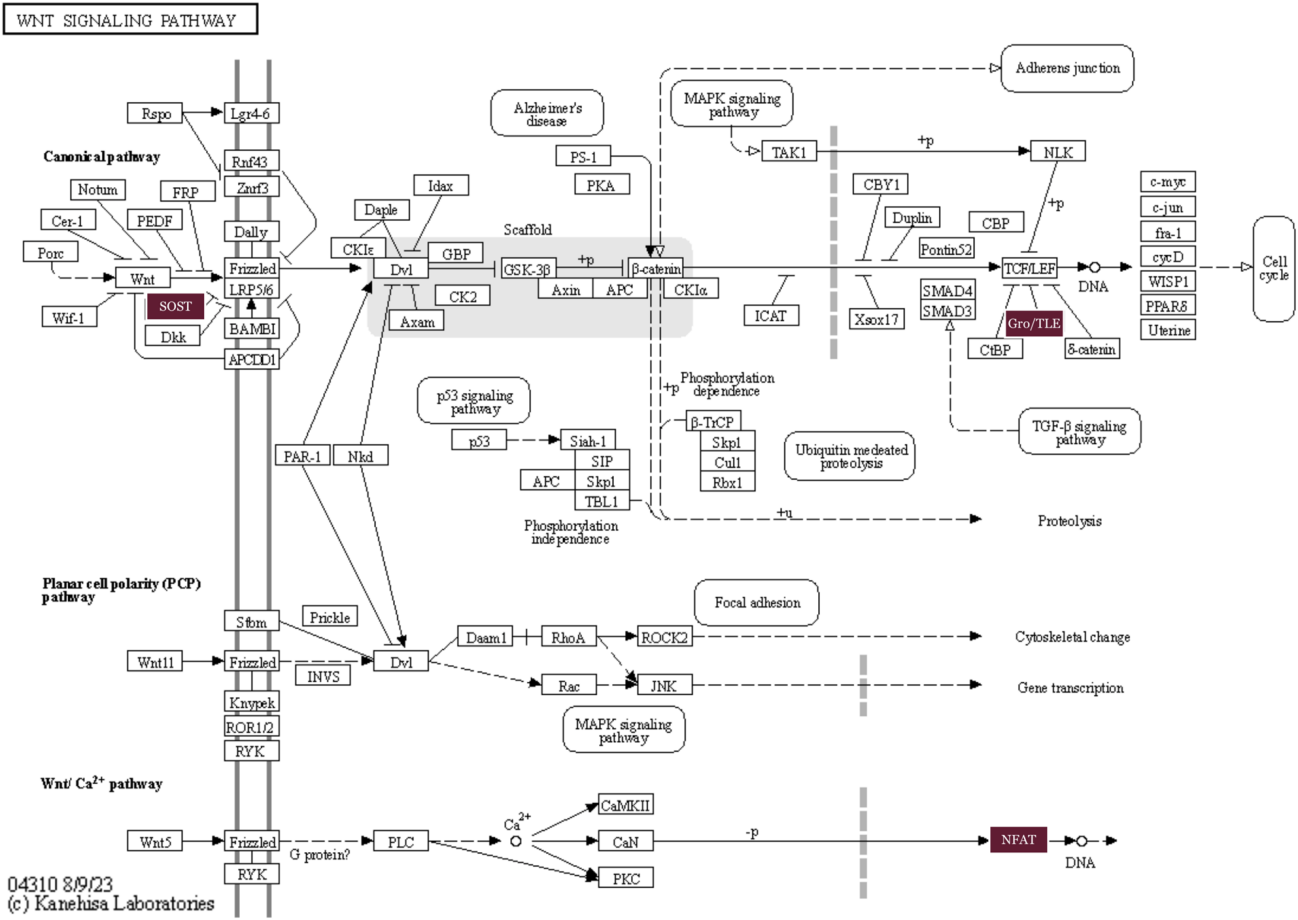
Wnt signaling pathway. The identified genes *SOST, Gro/TLE*, and *NFAT* are highlighted (R Shiny package ShineyGO, v0.77 & https://www.kegg.jp/pathway/hsa04310).

We ran a PPI network to further investigate the complex interactions between DMGs and find important hub proteins. A total of 606 PPI nodes of the hyper-methylated DMGs were constructed on the basis of the STRING database (Fig. 6, see R Shiny package ShinyGo^44^). The 16 node proteins, including *KIT, SEMA7A, BDNF, MEF2A, LDB2, GATA4, LHX2, SOST, CTLA4, NKX2-2, TLE4, BMP5, NFATC1, ZFPM1, DPYSL2,* and *ITGA2B* that showed a close interaction with other node proteins were chosen as hub genes (Fig. 7a, see Cytoscape^39^). The most important biological process and KEGG pathways of hub genes are shown in Fig. 7b and c. One important module was selected when the number of nodes is greater than 4. The key module demonstrated functions enriched in pathways such as Wnt signaling^58^ (Table 2 and Fig. 8, see R Shiny package ShinyGo^44^)).

**Figure 9:**
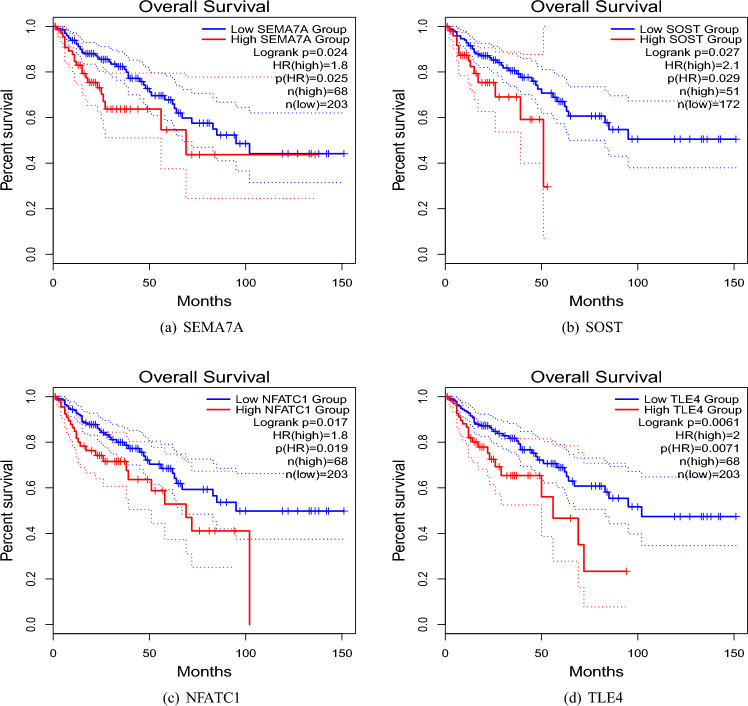
Overall survival of CRC patients stratified by their hub gene expression levels (http://gepia2021.cancer-pku.cn).

We performed a survival analysis using the TCGA-selected samples to investigate the association of selected hub genes with the survival time of CRC patients. Based on Fig. 9a–d (see GEPIA2021^59^), those patients with gene *SEMA7A* ($p=0.024$), *SOST* ($p=0.027$), *NFATC1* ($p=0.017$), and *TLE4* ($p=0.0061$) being upregulated, had a significantly lower probability of survival. However, this conclusion is based on univariate analysis, and the effect of other genes and the potential heterogeneity of DMG effects were ignored. We reanalyzed these data by accounting for the heterogeneity of DMG effects and obtained different results as follows.

##### Intangible heterogeneity of DMG effects on survival time

We studied the relationship between the average promoter methylation of the identified DMGs and the survival time subject to right-censoring by accounting for the heterogeneity of gene effects using an independent set of 521 TCGA CRC samples. To this end, we screened all the 1571 candidate DMGs using the correlation-adjusted regression survival scores to obtain the list of top candidate covariates. This process led to the selection of 95 highly correlated DMGs. These genes were also dysregulated in the TCGA samples. In addition, 4 hub genes that were related to the survival time of CRC patients were added to the list of covariates.

**Figure 10:**
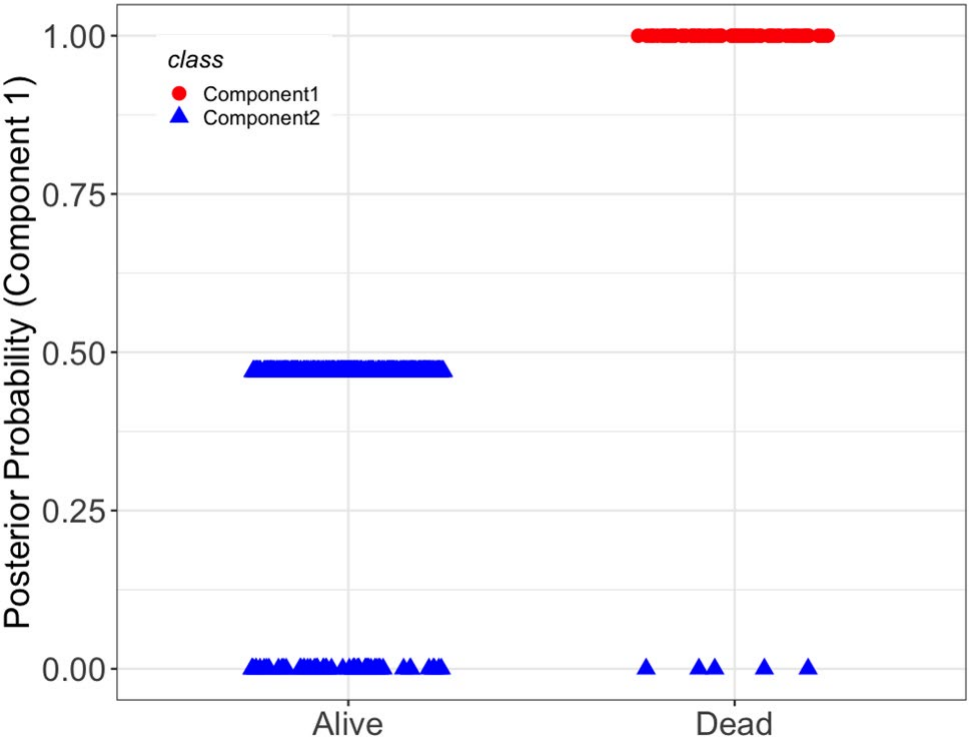
Posterior probability of CRC patients belonging to Component 1 separated for alive and deceased groups (R package fmrs, v2.0.1).

Our analysis yielded a two-component mixture of AFT regression model. The estimated gene effects on the survival time are given in Table 3. The result showed that 46% of the subjects were classified into Component 1, which is the most aggressive form of the disease. Figure 10 (see R package fmrs^60^) depicts the posterior probability of a subject belonging to Component 1. From this figure, we noticed that all living patients were classified into Component 2, which is the less aggressive form of the disease. A total of 83 and 18 DMGs were active in Components 1 and 2, respectively. Twelve genes including *HLA-F, MMP2, MT1A, RFPL4B, SIX6, ZFAT, BCKDK, AMOTL1, ADCY10, KCNK10, STAU2*, and *NOC4L* were not related to survival time in either of the components. These findings demonstrate the heterogeneity of DMG effects in CRC data and justify using a sparse mixture modeling rather than a univariate one. In addition, the DMGs with active promoters in Component 1 can be considered biomarkers for CRC prognosis. The bioinformatics and biological information of selected DMGs are given in Table 4.

**Table 3:** Estimated DMG effects in the two-component mixture of accelerated failure time regression model in the CRC data.

Gene	$\beta _1$	$\beta _2$	Gene	$\beta _1$	$\beta _2$	Gene	$\beta _1$	$\beta _2$
*NMI*	− 27.2	0.0	*SIX6*	0.0	0.0	*FOXF2*	− 12.1	0.0
*NCOA4*	− 13.7	96,804.5	*FOXP2*	12.6	− 101,775.9	*GIPR*	− 19.0	0.0
*ANKMY1*	− 32.6	0.0	*TNFSF9*	− 14.7	0.0	*UCKL1*	− 45.0	0.0
*ST6GAL2*	6.2	0.0	*CLDN3*	− 2.1	21,941.6	*AMOTL1*	0.0	0.0
*PSMG3*	− 12.4	− 28,758.9	*DDX46*	40.0	0.0	*GMPS*	− 6.8	0.0
*FAR2*	− 21.6	0.0	*ZFAT*	0.0	0.0	*ADCY10*	0.0	0.0
*MPPED2*	− 14.7	0.0	*OR5M1*	− 6.0	0.0	*GPM6A*	− 18.6	0.0
*GTF2IRD1*	− 14.5	0.0	*PHACTR3*	6.3	0.0	*PFKP*	2.6	0.0
*FKBP6*	− 11.9	0.0	*KRTAP13-4*	4.7	− 15,847.1	*C14orf39*	2.4	− 15,364.8
*SNORD109B*	− 6.5	0.0	*LOC400940*	− 6.6	70,576.5	*KCNK10*	0.0	0.0
*HLA-F*	0.0	0.0	*LRTM1*	− 13.4	− 50,609.5	*STK32B*	18.4	0.0
*AKAP9*	7.1	0.0	*NPAS2*	125.0	0.0	*IL1A*	13.3	0.0
*SEMA4F*	− 21.3	0.0	*AXIN2*	24.3	0.0	*KRTAP20-1*	5.0	0.0
*RPL23P8*	18.1	0.0	*NKX2-3*	0.0	− 13,689.5	*KIRREL2*	− 13.1	0.0
*CHI3L1*	4.6	0.0	*NT5M*	18.8	0.0	*C1D*	− 28.8	0.0
*NCAN*	3.7	151,828.5	*MECOM*	44.5	0.0	*EGR2*	54.7	0.0
*CLEC5A*	− 10.4	0.0	*LUZP6*	− 73.9	0.0	*PDF*	− 1.4	8045.7
*TRPS1*	− 16.7	0.0	*FLJ16779*	0.0	− 87,806.8	*KCNQ3*	23.4	0.0
*CMKLR1*	18.1	0.0	*SLC25A24*	− 6.0	− 77,923.0	*CCR5*	− 20.3	0.0
*GABRA4*	− 6.2	0.0	*C1QTNF7*	− 10.6	0.0	*COL4A3*	0.0	34,903.1
*OR5AS1*	− 39.6	0.0	*MTNR1B*	11.7	0.0	*TFAP2C*	7.9	0.0
*MMP2*	0.0	0.0	*NMNAT2*	− 12.0	0.0	*GNG2*	7.1	0.0
*AKAP12*	6.6	0.0	*BCKDK*	0.0	0.0	*OC90*	0.8	80,377.8
*PSD2*	5.4	− 82,538.6	*ZFP42*	− 13.8	0.0	*LHFPL2*	21.5	0.0
*FGFR1*	14.0	0.0	*CALB1*	− 5.9	0.0	*STAU2*	0.0	0.0
*KIRREL3*	− 10.3	0.0	*TCHH*	− 17.8	0.0	*OLFM3*	10.3	0.0
*HECA*	− 6.8	0.0	*MAPT*	− 14.1	0.0	*SLTM*	− 133.5	0.0
*MT1A*	0.0	125,763.0	*SYDE1*	4.2	− 364,254.7	*NOC4L*	0.0	0.0
*NUDT13*	− 7.3	0.0	*RNASE3*	7.0	0.0	*CNDP2*	0.0	0.0
*STON1-GTF2A1L*	− 21.3	0.0	*PLCD3*	58.7	0.0	*NFATC1*	− 20.3	0.0
*LBP*	− 7.7	0.0	*MAP1LC3A*	5.8	0.0	*SEMA7A*	21.4	0.0
*MYLK3*	21.9	0.0	*CROCC*	18.2	0.0	*SOST*	− 2.5	0.0
*RFPL4B*	0.0	0.0	*OPCML*	21.4	0.0	*TLE4*	− 7.2	0.0

**Table 4:** Bioinformatics and biological information of selected DMGs related to survival in colorectal cancer patients.

Gene	Information	Summary
*CLDN3*	Description	Claudin 3
	Predicted location	Membrane
	Protein class	Cancer-related genes, Disease-related genes, Potential drug targets, Transporters
	Cell line specificity	Cancer enhanced (CRC)
	Pathway	Cell adhesion tight junctions, Cell adhesion endothelial cell contacts by junctional mechanisms
	Function	Contributes to the closure of intercellular gaps within tight junctions through calcium-independent cell adhesion.
	Cancer	Tends to be down-regulated in primary CRC samples and can predict prognosis in CMS2 or CMS3 CRC subtypes.
	Reference	Perez et al.^61^, Cherradi et al.^62^
*NFATC1*	Description	Nuclear Factor of Activated T Cells 1
	Predicted location	Intracellular
	Protein class	Transcription factors
	Cell line specificity	Cancer enhanced (Lymphoma)
	Pathway	Activation of cAMP-dependent PKA, Activation of PKA through GPCR, APRIL pathway, BAFF in B-Cell signaling, cAMP pathway
	Function	Contributes to the inducible expression of cytokine genes in T-cells, influencing the transcription of genes like IL-2 and IL-4. It also affects gene expression in embryonic cardiac cells, and plays a role in T-lymphocyte activation, proliferation, differentiation, and programmed cell death.
	Cancer	Activates the transcription of SNAI1, facilitating EMT and CRC metastasis. It’s an immune-related prognostic risk factor for CRC immunotherapy.
	Reference	Chuvpilo et al.^63^, Shen et al.^64^, Wu et al.^65^
*AXIN2*	Description	Axin 2
	Predicted location	Intracellular
	Protein class	Cancer-related genes, Disease-related genes, Plasma proteins
	Cell line specificity	Cancer enhanced (CRC, Gastric cancer)
	Pathway	Wnt signaling pathway, Cytoskeleton remodeling reverse signaling by ephrin B
	Function	Plays a role in stabilizing beta-catenin within the Wnt signaling pathway, similar to mouse conductin and rat axil in rodents.
	Cancer	AXIN1/2 alterations may be key defects in some cancers including CRC and hepatocellular carcinoma.
	Reference	Mazzoni et al.^66^
*SEMA7A*	Description	Semaphorin 7A
	Predicted location	Membrane
	Protein class	Disease-related genes
	Cell line specificity	Low cancer specificity
	Pathway	Axon guidance, Developmental biology, Nervous system development, Other semaphorin interactions, Semaphorin interactions
	Function	Has a significant role in integrin-mediated signaling, governing cell migration and immune reactions. Facilitates the assembly of focal adhesion complexes, triggers the activation of protein kinase PTK2/FAK1, leading to MAPK1 and MAPK3 phosphorylation.
	Cancer	Associated with Breast, Lung, and Pancreatic cancers.
	Reference	Mastrantonio et al.^67^, Fijneman et al.^68^, Liu et al.^69^
*UCKL1*	Description	Uridine-Cytidine Kinase 1 Like 1
	Predicted location	Intracellular
	Protein class	Enzymes, Metabolic proteins
	Cell line specificity	Low cancer specificity
	Pathway	Pyrimidine metabolism
	Function	Encodes a uridine kinase, converting uridine into uridine monophosphate. Its ubiquitination increases with natural killer lytic-associated molecule presence, resulting in protein degradation. A potential therapeutic target for inhibiting tumor growth and metastasis.
	Cancer	A candidate gene in CRC.
	Reference	Long et al.^70^, Matchett et al.^71^
*ANKMY1*	Description	Ankyrin Repeat and MYND Domain containing 1
	Predicted location	Intracellular
	Protein class	–
	Cell line specificity	Low cancer specificity
	Pathways	WP5224 pathway
	Function	Predicted to enable metal ion binding activity.
	Cancer	Associated with Osteosarcoma.
	Reference	Wang et al.^72^
*TLE4*	Description	TLE family member 4, transcriptional corepressor
	Predicted location	Intracellular
	Protein class	–
	Cell line specificity	Low cancer specificity
	Pathway	Wnt signaling pathway, Development Notch signaling pathway
	Function	Transcriptional corepressor that binds to various transcription factors. Inhibits transcriptional activation by PAX5, CTNNB1, and TCF family members in the Wnt signaling pathway.
	Cancer	Overexpression may play a role in CRC development and progression, partly through the JNK/c-Jun pathway. It is a candidate for risk stratification of cancer recurrence after curative resection of early-stage CRC.
	Reference	Wang et al.^73^, Yu et al.^74^
*EGR2*	Description	Early Growth Response 2
	Predicted location	Localized to the Nucleoplasm
	Protein class	Disease related genes, Transcription factors
	Cell line specificity	Cancer enhanced (Lymphoma)
	Pathway	Activation of anterior HOX genes in hindbrain development during early embryogenesis, Activation of HOX genes during differentiation
	Function	Mutations linked to Charcot–Marie–Tooth disease type 1D, Charcot–Marie–Tooth disease type 4E, and Dejerine-Sottas syndrome.
	Cancer	Targeting EGR2 may provide a therapeutic strategy to eliminate colon cancer stem cells and block nervous system-driven disease progression through differentiation.
	Reference	Regan et al.^75^
*SLTM*	Description	SAFB Like Transcription Modulator
	Predicted location	Intracellular
	Protein class	–
	Cell line specificity	Low cancer specificity
	Pathway	–
	Function	Hypothesized to play a role in mRNA processing regulation and RNA polymerase II-mediated transcription regulation.
	Cancer	Up-regulated in dextran sulfate sodium treated colon mucosa.
	Reference	De Robertis et al.^76^
*PLCD3*	Description	Phospholipase C Delta 3
	Predicted location	Intracellular
	Protein class	Enzymes, Metabolic proteins, Plasma proteins
	Cell line specificity	Low cancer specificity
	Pathway	Wnt signaling pathway
	Function	Crucial for trophoblast and placental development, possibly contributing to cytokinesis by cleavage furrow PIP2 hydrolysis. Controls neurite outgrowth by suppressing RhoA/Rho kinase signaling.
	Cancer	Down-regulation of Phosphatidylinositol signaling system pathway in CRC mucosa.
	Reference	Danielsen et al.^77^
*MAPT*	Description	Microtubule Associated Protein Tau
	Predicted location	Intracellular
	Protein class	Disease related genes, FDA approved drug targets, Plasma proteins
	Cell line specificity	Group enriched (Bone cancer, Neuroblastoma)
	Pathway	AMPK signaling pathway, P38 MAPK signaling pathway
	Function	Promotes microtubule assembly and stability, maintaining neuronal polarity. Binds axonal microtubules and neural plasma membrane components, acting as a link between them. Its localization within the cell body helps define axonal polarity.
	Cancer	Hyper-methylation in MAPT is associated with poor prognosis in stage II CRC patients.
	Reference	Sandberg et al.^78^, Wang et al.^79^
*FOXF2*	Description	Forkhead box F2
	Predicted location	Intracellular
	Protein class	Transcription factors
	Cell line specificity	Low cancer specificity
	Pathway	–
	Function	Among human counterparts of Drosophila melanogaster forkhead transcription factor. Expresses in the lung and placenta, activating transcription of several lung-specific genes.
	Cancer	Regulates PRUNE2 transcription in CRC pathogenesis and is hyper-methylated in CRC samples.
	Reference	Li et al.^80^, Hauptman et al.^81^
*TNFSF9*	Description	TNF superfamily member 9
	Predicted location	Intracellular
	Protein class	Plasma proteins
	Cell line specificity	Cancer enhanced (Kidney cancer)
	Pathway	Cytokine signaling in immune system, TNFR2 non-canonical NF-kB pathway
	Function	Cytokine in the TNF ligand family, acting as a bidirectional signal transducer with TNFRSF9/4-1BB. Key role in antigen presentation, cytotoxic T cell generation, and T lymphocyte activation and proliferation.
	Cancer	Critical role in liver homing for metastatic colon cancer.
	Reference	Barderas et al.^82^

## Discussion

Colorectal cancer is one of the deadliest cancers in the world. Given that early stages of CRC do not display symptoms, proactive screening is the only viable approach to identify the disease^83^. As DNA methylation changes are closely associated with cancer, their role in CRC biomarker detection in the early stages of cancer is of great importance. Although many CRC biomarkers have been detected in the literature, only a few are used in practice. Our findings resulted in identifying new biomarkers for CRC which can be used for diagnosis and prognosis.

We identified 1,571 DMGs most of which have been previously studied in the literature. Among them, *SEPT9, NDRG4, VIM, APC, SFRP1, SFRP4*, and *SFRP5*,^84^ are the most important CRC-related ones. We also explored CRC-related hub genes. Fourteen functional modules that may play important roles in the early detection of CRC were highlighted and the sub-network of hub genes *KIT, SEMA7A, BDNF, MEF2A, LDB2, GATA4, LHX2, SOST, CTLA4, NKX2-2, TLE4, BMP5, NFATC1, ZFPM1, DPYSL2*, and *ITGA2B* was extracted. These hub genes were flagged as potential diagnostic and therapeutic targets for CRC in our analysis.

In addition to the diagnostic role of our identified hub genes such as *NKX2-2, KIT, BNDF*, and *TLE4* in CRC and its sub-types^74,85–87^, their roles in increasing CRC risk, tumor progression, and targeted therapy have been investigated. For instance, *MEF2A*^88^ and *BMP5*^89^ increase the CRC risk. Up-regulation of the expression of *ITGB7* and *ITGA2B* has been found to be significantly associated with death by sodium butyrate-induced CRC organoids^90^. Moreover, some studies^91,92^ have shown effective treatments by targeting *CLT-4* and *LDB2n*.

There is a rich literature on the contribution of some of our identified hub genes in CRC and less evidence in support of some others such as *LHX2, ZFPM1*, and *DPYSL2*. For instance, the differences in tumor and corresponding adjacent benign tissues regarding *LHX* gene expressions have been investigated^93^. However, contrary to our findings, they did not find any statistical differences for *LHX2* and *LHX3* genes. Furthermore, the upregulation of *ZFPM1* was revealed in molecular high-risk patients with cytogenetically normal acute myeloid leukemia^94^, yet its diagnostic value in CRC has not fully been confirmed^95^. *SEMA7A* is also one of our selected hub genes that play a key role in several cancers including pancreatic, breast, and lung cancers^69,96–98^. However, there has been less attention on the role of *SEMA7A* in CRC. Further investigation is required on our flagged DMGs.

Although there are many mechanisms that drive CRC, only a handful of them have been discovered in past studies. As researchers continue to genotype large panels of CRC tumors, it can be expected that additional new pathways of CRC carcinogenesis will be revealed. *SOST*, an identified hub gene in our study, plays a vital role in inhibiting the Wnt signaling pathway by binding to the Wnt co-receptor, LRP5/6, and preventing its activation^99^. Therefore, decreased *SOST* expression could lead to an increase in Wnt signaling, promoting CRC cell proliferation, migration, and survival. Another identified hub gene is *TLE4* which is involved in the negative regulation of the canonical Wnt signaling pathway. Only a few investigations provided evidence of *TLE4* upregulation in CRC biopsies, partially through regulation of the JNK/c-Jun pathway^73^. Moreover, recent studies that focus on the NFAT signaling pathway showed a promising strategy for CRC treatment^64^.

Heterogeneity is one of the key features of genomic data. Specifically, there is evidence of the heterogeneity of DMG effects on the survival of CRC patients in the literature and in our dataset. The finite mixture of the AFT regression model is a plausible method to uncover such intangible heterogeneity. Our analysis suggested a mixture of two-component mixture of the AFT regression model in which patients were separated into two subgroups based on their vital status. In this model, almost all of the deceased patients were classified into the most aggressive form of the disease (Component 1). In Component 1, 83 DMGs including *NMNAT2, ZFP42, NPAS2, MYLK3, NUDT13, KIRREL3*, and *FKBP6* had an effect on the survival time of the patients. The relation between some of these DMGs and survival time has been previously reported^100^. On the other hand, there are a few discoveries regarding other genes. For instance, significantly higher expression of *NMNAT2* in CRC tissues compared to normal ones have been found, yet this gene was not a prognostic factor for overall survival^101^. Note that, while the hub genes *SOST, NFATC1*, and *TLE4* were associated with survival in the univariate Cox model, they were only associated with survival time in the most aggressive form of the disease in our study.

Our study does not exclusively depend on bioinformatics analysis, as we have employed several statistical and machine learning analyses. These include modeling methylation profiles, identifying DMCs via DMCHMM, conducting statistical tests, performing multiple validation analyses, and applying statistical learning algorithms to survival times via fmrs. One of the advantages of the DMCHMM method is that it does not require a large number of samples or matched samples, as it is highly flexible and can accommodate various experimental designs. It demonstrates significant power, particularly when dealing with moderate to low sample sizes.

### Supplementary Information

Supplementary Information.

## Data Availability

In this study methylation profiling datasets with accession numbers GSE53051, GSE77718, GSE101764, GSE42752, and GSE48684 were obtained from Gene Expression Omnibus (GEO, https://www.ncbi.nlm.nih.gov/geo/), of the National Center for Biotechnology Information (NCBI). Additional DNA methylation datasets and expression profiles of CRC patients (TCGA-COAD, TCGA-READ, TCGA-SARC projects) were obtained from The Cancer Genome Atlas (TCGA, https://www.cancer.gov/ccg/research/genome-sequencing/tcga), of the National Cancer Institute (NCI). Our SureSelectXT Human Methyl-Seq dataset on methylation profiles of 6 patients with adenocarcinoma of CRC and 6 normal males is obtained from ‘Reza Radiotherapy and Oncology Center’ in Iran and is available upon request.
